# Microbe-Mediated Activation of Toll-like Receptor 2 Drives PDL1 Expression in HNSCC

**DOI:** 10.3390/cancers13194782

**Published:** 2021-09-24

**Authors:** Jacqueline E Mann, Megan L Ludwig, Aditi Kulkarni, Erin B Scheftz, Isabel R Murray, Jingyi Zhai, Elizabeth Gensterblum-Miller, Hui Jiang, J Chad Brenner

**Affiliations:** 1 Department of Otolaryngology—Head and Neck Surgery, University of Michigan, Ann Arbor, MI 48109, USA; jacmann@umich.edu (J.E.M.); ludwigml@umich.edu (M.L.L.); aditisk@umich.edu (A.K.); escheftz@umich.edu (E.B.S.); irmurray@umich.edu (I.R.M.); gensterb@umich.edu (E.G.-M.); 2 Department of Pathology, University of Michigan, Ann Arbor, MI 48109, USA; 3 Cellular and Molecular Biology Program, University of Michigan, Ann Arbor, MI 48109, USA; 4 Department of Biostatistics, University of Michigan, Ann Arbor, MI 48109, USA; jyzhai@umich.edu (J.Z.); jianghui@umich.edu (H.J.); 5 Rogel Cancer Center, University of Michigan, Ann Arbor, MI 48109, USA; 6 Department of Pharmacology, University of Michigan, Ann Arbor, MI 48109, USA

**Keywords:** CRISPR, TLR2, microbiome, HNSCC, PDL1

## Abstract

**Simple Summary:**

Tumors use immunosuppressive signals to evade detection by the immune system. While recurrent and metastatic head and neck squamous cell carcinoma has historically carried a poor prognosis, therapies targeting the immunosuppressive PD1:PDL1 axis have improved survival in certain patients. Defining mechanisms regulating PDL1 in various contexts may inform refinement of immunotherapy protocols. We identified a role for Toll-like Receptor 2 (TLR2) signaling in driving PDL1 expression. In antigen-presenting cells, TLR2 functions to initiate response to pathogens, and it is overexpressed or genetically altered in some tumors. We found that the synthetic TLR2 ligand Pam3CSK4, as well as whole bacteria, induced PDL1 expression in specific HNSCC cell line models, suggesting that TLR2 may contribute to immune evasion in chronically inflamed tissues.

**Abstract:**

As immunotherapies targeting the PDL1 checkpoint have become a mainstay of treatment for a subset of head and neck squamous cell carcinoma (HNSCC) patients, a detailed understanding of the mechanisms underlying PDL1-mediated immune evasion is needed. To elucidate factors regulating expression of PDL1 in HNSCC cells, a genome-wide CRISPR profiling approach was implemented to identify genes and pathways conferring altered PDL1 expression in an HNSCC cell line model. Our screen nominated several candidate PDL1 drivers, including Toll-like Receptor 2 (TLR2). Depletion of TLR2 blocks interferon-γ-induced PDL1 expression, and stimulation of TLR2 with either *Staphylococcus aureus* or a bacterial lipopeptide mimetic, Pam3CSK4, enhanced PDL1 expression in multiple models. The data herein demonstrate a role for TLR2 in modulating the expression of PDL1 in HNSCC models and suggest that microbiota may directly modulate immunosuppression in cancer cells. Our study represents a step toward disentangling the diverse pathways and stimuli regulating PDL1 expression in HNSCC and underscores a need for future work to characterize the complex microbiome in HNSCC patients treated with immunotherapy.

## 1. Introduction

Head and Neck Squamous Cell Carcinoma (HNSCC) is newly diagnosed in 600,000 patients worldwide each year [1]. HNSCCs are often associated with either a history of alcohol and tobacco use or human papillomavirus infection and have overall 5-year survival rates ranging from 40 to 75%, depending on sub-site [2]. While survival in this population remained relatively unchanged over the past several decades, immune checkpoint blockade has revolutionized therapy for HNSCC patients, with up to 20% of recurrent or metastatic patients responding to immunotherapies targeting the PD1:PDL1 interaction [3]. Pembrolizumab and nivolumab, both anti-PD1 antibodies, were shown to improve upon investigator’s choice of chemotherapy in patients with platinum-refractory HNSCC and were approved for first-line use in this population in 2019 [4,5]. Further, the KEYNOTE-048 trial demonstrated that first-line pembrolizumab monotherapy was superior to cetuximab with chemotherapy in recurrent/metastatic HNSCC patients whose tumors express PDL1 [6]. Given the success of these strategies in subsets of patients, and the evidence for PDL1 expression predictive biomarker, we sought to understand the mechanisms regulating this checkpoint and to discover factors that may contribute to response or failure of PD1 blockade.

Cell-surface PDL1 expression in tumors is induced by interferon gamma (IFNγ), a cytokine released by T-lymphocytes, and relies on JAK2/STAT1 mediated transcription in HNSCC cells [7]. In HNSCC cell lines, activation of epidermal growth factor receptor (EGFR), an established oncogenic driver and therapeutic target in HNSCC, was shown to positively regulate PDL1 expression on the cell surface [8]. These observations led us to question whether additional pathways, including oncogenic signals and external stimuli in the microenvironment, may contribute to immune escape via PDL1 upregulation.

In recent years, new applications for genome-scale functional screening have rapidly arisen alongside advances in CRISPR technology [9,10]. Here, we sought to leverage this technology to define genes and pathways involved in the regulation of cell-surface expression of PDL1 in HNSCC.

## 2. Materials and Methods

### 2.1. Cell Culture

Cell lines were maintained in logarithmic growth phase in Dulbecco’s Modified Eagle’s Medium (Gibco, Amarillo, TX, USA) containing 10% fetal bovine serum (FBS; Sigma, St. Louis, MO, USA), 1% NEAA (Gibco), and 100 U/mL penicillin-streptomycin (Gibco) in a humidified atmosphere of 5% CO_2_ at 37 °C. Cells were tested for mycoplasma contamination using the MycoAlert detection kit (Lonza, Basel, Switzerland). All models were genotyped as previously described [11].

### 2.2. PDL1 Induction

Where indicated, cells were treated with 10 ng/mL IFNγ (R&D Systems, Minneapolis, MN, USA, #285-IF), 500 ng/mLPam3CSK4 (Invivogen, San Diego, CA, USA, #tlrl-pms), 0.075% heat-killed *Staphylococcus aureus* (Millipore, Burlington, MA, USA, #507858), 10^8^ CFU/mL heat-killed *Streptococcus pneumoniae* (Invivogen #tlrl-hksp), 10^8^ CFU/mL heat-killed *Listeria monocytogenes* (Invivogen #tlrl-hklm), 10 ug/mL heat-killed *Mycobacterium tuberculosis* (Invivogen #tlrl-hkmt-1) or 10^8^ CFU/mL heat-killed *Staphylococcus aureus* (Invivogen #tlrl-hksa). Invivogen products were rehydrated in endotoxin-free water per manufacturer instructions.

### 2.3. Transduction

UM-SCC-49 was transduced with the Human GeCKO CRISPR knockout pooled library version 2A in the lentiCRISPRv2 backbone (Addgene, Watertown, MA, USA, #52961). Conditions for transduction of the genome-wide gRNA library were established for a multiplicity of infection (MOI) of 0.3. Cells were subjected to 7 days of puromycin selection, then expanded and seeded for treatment and flow cytometry.

### 2.4. Fluorescence-Activated Cell Sorting

To preserve at least 300× library coverage, 30 million cells were seeded per treatment. Cells were treated with 10 ng/mL IFNγ (R&D Systems) for 72 h incubation, after which 30 million cells were incubated with a PDL1 monoclonal antibody (ThermoFisher #14-5983-82, 1 ug/mL in HBSS) for 15 min in a suspension of 1 million cells/mL followed by washing in PBS. Control (no primary antibody) and PDL1 stained cells were incubated with PE-conjugated rat anti-mouse secondary antibody (ThermoFisher #12-4015-82, 0.2 ug/mL in HBSS) for 15 min. Cell suspensions in HBSS supplemented with 0.5% FBS were gated on live cells and sorted on an iCyt Synergy (Sony Biotechnology, San Jose, CA, USA) at the University of Michigan Flow Cytometry Core.

### 2.5. GeCKO Library Preparation

Genomic DNA was extracted from the UM-SCC-49 GeCKO pool cells using the Gentra Puregene Cell Kit (Qiagen, Hilden, Germany). For each sample, 13 reactions with 10 μg genomic DNA input each were used to PCR amplify gRNA sequences using Herculase II Fusion DNA Polymerase (Agilent, Santa Clara, CA, USA, #600675). Sequencing adapters and barcodes were added to the PCR products using primers listed in Table S1. Amplicons were purified using PCR Purification Kit (Qiagen) before submission to the University of Michigan DNA Sequencing Core for sequencing with Illumina MiSeq V3 Kit.

### 2.6. Analysis of CRISPR Libraries

Reads were demultiplexed by barcode and then mapped to the corresponding reference library using an in-house python script. gRNAs represented by fewer than ten reads were excluded from further analyses. Read counts were normalized to the total number of reads for a given sample and the read count for each gRNA was then computed relative to the read count in the control to determine relative abundance in the sorted populations versus control (unsorted).

### 2.7. SiRNA Transfection

ON-TARGETplus siRNA SMARTpools were purchased from Horizon Discovery (Cambridge, UK; TLR2, L-005120-01-0005; RELA, L-003533-00-0005; GAPD, L-004253-00-0005). Non-targeting siRNA was purchased from Horizon Discovery (D-001810-02-05). Transfections were performed 18 h post seeding using oligofectamine (Invitrogen, Carlsbad, CA, USA, #12252011) per manufacturer’s protocol. For qPCR analysis, RNA was isolated 24 h after initial transfection. For analysis of PDL1 protein, cells were treated as indicated for an additional 48 h, then total protein lysate was collected.

### 2.8. Western Blotting

Cells were rinsed twice with ice cold PBS and lysed in a modified RIPA lysis buffer (150 mM NaCl, 50 mM Tris pH 8.0, 1 mM PIPES, 1 mM MgCl, 10% Glycerol, 1% NP40, 0.1% Triton X-100) with HALT protease and phosphatase inhibitor cocktails (Thermo Scientific #186129, 1861277). Separation by SDS-PAGE was performed and the following antibodies purchased from Cell Signaling Technology (Danvers, MA, USA) were used for visualization of target proteins: PDL1 (#13684), β-actin (#4970), NFκB p65 (#8242), phospho(Ser-536)-NFκB p65 (#3033).

### 2.9. qPCR

RNA extraction and cDNA synthesis using SuperScript III Reverse Transcriptase VILO kit (Invitrogen) was performed according to manufacturer protocols using primers in Table S2. Amplification by qPCR was performed with QuantiTect SYBR Green (Qiagen) on QuantStudio5 (Applied Biosystems, Waltham, MA, USA) under the cycling conditions recommended by the manufacturer.

## 3. Results

**Figure 1:**
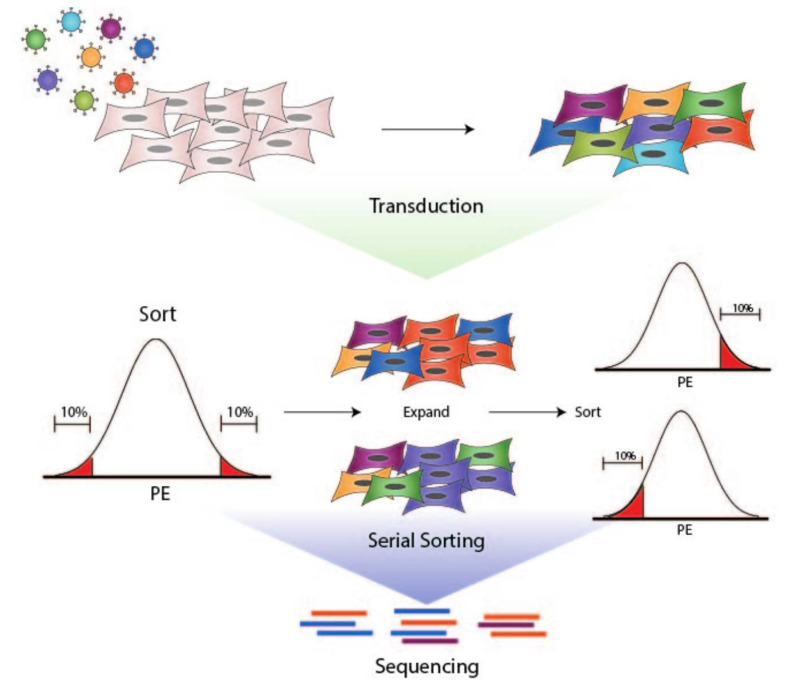
Workflow for generation of UM-SCC-49-GeCKO pool, cell sorting, and sequencing of final populations. 1. UM-SCC-49 was transduced with a lentiviral pooled sgRNA library (GeCKO v2). 2. Live cells were selected based on PDL1 expression using a PE-conjugated antibody directed against PDL1. PE fluorescence distribution was plotted on a histogram. The fractions of cells with the highest and lowest PE fluorescence (10% each) were expanded separately in culture and re-sorted to enhance respective phenotypes. 3. DNA was harvested from final populations for next-generation sequencing of sgRNA barcodes.

**Figure 2:**
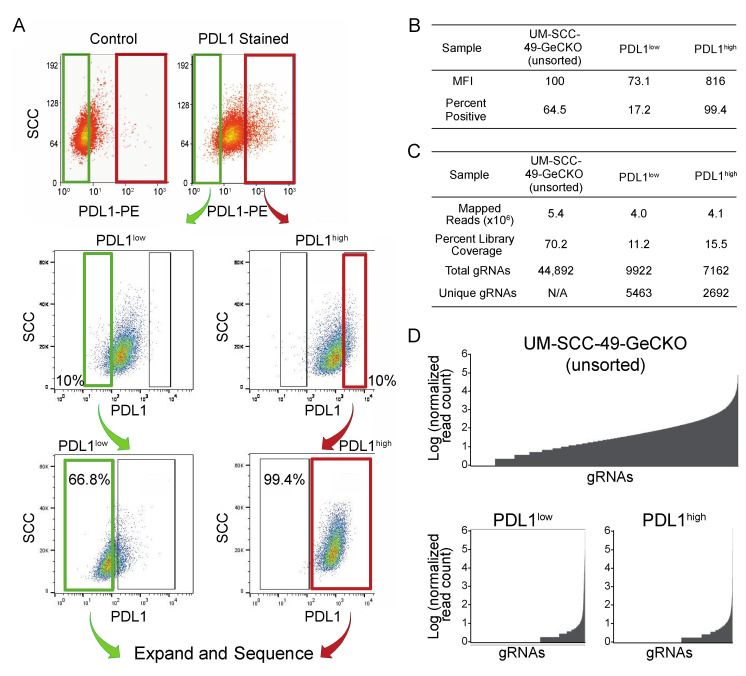
Sorting of UM-SCC-49-GeCKO pool for PDL1 enhanced or deficient cells. (**A**) UM-SCC-49 cells infected with the GeCKO library were treated with 10 ng/mL IFNγ for 72 h and stained using a PE-conjugated antibody directed against PDL1. Cells were then subjected to fluorescence-activated cell sorting. Gates were drawn to select the 10% of cells with lowest fluorescence (PDL1^low^; upper plots) and 10% with the highest (PDL1^high^; upper plots). Green and red gates indicate populations of cells collected for PDL1^low^ and PDL1^high^, respectively. The selected populations were expanded separately in culture to 30 million cells each, then subjected to cell sorting again, selecting the lowest 10% of cells from PDL1^low^ and the highest 10% of cells from PDL1^high^ (middle plots). Again, these cells were expanded and analyzed by flow cytometry a third time to ensure divergent phenotypes. “positive” and “negative” gates were drawn based on 0.1% and 99% (respectively) of a control stained with secondary antibody only (lower plots). At this point, all PDL1-negative (PDL1^low^) and all PDL1-positive (PDL1^high^) cells were selected, and genomic DNA was harvested. (**B**) Median fluorescence intensities (MFIs) and percent of cells positive for PDL1 are reported for the final sort for each population. (**C**) Mapping statistics from UM-SCC-49-GeCKO sequencing results. (**D**) Distribution of read counts across gRNAs (normalized to total reads for each sample and log transformed).

We developed a genome-wide CRISPR screen to identify genes that modulate cell surface PDL1 expression using a positive selection strategy to enrich for cells with high or low PDL1 expression following IFNγ stimulation (Figure 1). UM-SCC-49, which was derived from an aggressive tongue HNSCC, was selected as a model based on its ability to upregulate PDL1 in response to IFNγ compared to other HNSCC cell lines (data not shown), previously published exome sequencing demonstrating that genes from the established IFNγ signaling pathway are not mutated in this cell line, and growth characteristics amenable to scaling cell culture for high throughput screening [12]. UM-SCC-49 cells were transduced with the GeCKO v2A CRISPR library, treated with IFNγ, and serially sorted to collect populations exhibiting the highest (PDL1^high^) and lowest (PDL1^low^) IFNγ-induced PDL1 expression (Figure 2A). After expanding the final population, we showed divergence between PDL1^high^ and PDL1^low^ phenotypes as indicated by the median fluorescence intensity (MFI) of each population and/or the percentage of cells positive for PDL1 expression (Figure 2A,B). Clonal cell lines from single cells isolated from the PDL1^low^ pool exhibited diminished ability to upregulate total PDL1 protein expression in response to IFNγ, indicating that we had selected individual cells with dysregulated PDL1 (Figure S1).

Barcodes from the initial UM-SCC-49 GeCKO library pool (control) and the sorted PDL1^high^ and PDL1^low^ pools were sequenced and quantified to identify enrichment of specific knockouts. In the control pool, 44,892 gRNAs were identified (70% library coverage), while 9822 gRNAs were identified in PDL1^high^ (16% library coverage) and 7162 in PDL1^low^ (11% library coverage; Figure 2C,D). These changes in library coverage and gRNA distribution support the selection of specific gRNA subsets.

For our analysis, we chose a normalized read count cutoff of greater than or equal to 10 gRNAs and defined a rank list of hits for which representation was uniquely enriched over the control pool. gRNAs enriched >1.5× over the control in either sorted population are reported in Table 1 (PDL1^low^) and Table 2 (PDL1^high^). Importantly, we observed an interesting relationship between some of the most enriched gRNAs in the PDL1^high^ and PDL1^low^ pools. We identified a gRNA targeting Toll Like Receptor 2 (TLR2) among the most abundant in the PDL1^low^ pool, while gRNAs targeting mir-105-1 and mir-105-2, which may negatively regulate toll like receptors [13], were enriched in PDL1^high^ population. TLRs are a widely studied class containing 9 different type I transmembrane proteins that share similar domain structures, with a leucine-rich extracellular domain, transmembrane domain and cytosolic toll/interleukin-1 receptor domain [14]. When activated by microbial ligands, TLRs transmit signals through overlapping signaling cascades, often via Myd88 and NFκB to promote innate and adaptive immune responses [15]. Although TLRs, including TLR2, are expressed in mucosal epithelia and are overexpressed or genetically altered in numerous tumor types, a mechanistic link between TLR activation and evasion of anti-tumor immunity has not been thoroughly explored. Therefore, TLR2 represented a promising novel target for further evaluation in HNSCC cells.

Given the enrichment of gRNAs targeting TLR2 and potential TLR2-modulating miRNAs, we hypothesized that TLR2 may positively regulate PDL1 expression in the UM-SCC-49 model. We therefore examined whether genes nominated by our screen were co-expressed with CD274 (encoding the PDL1 protein) in HNSCC tumors using publicly available RNAseq data from the Cancer Genome Atlas HNSCC project (*n* = 566, UCSC cancer genomics browser (xenabrowser.net), accessed on 4 October 2019; Table 3). As proof of concept, we showed that expression of JAK2, an established driver of CD274 transcription [7,8], correlates significantly with CD274 expression in HNSCC (Pearson r = 0.69, *p* < 0.0001; Figure S2A). We discovered a significant positive correlation between TLR2 and CD274 expression (Pearson r = 0.32, *p* < 0.0001; Figure S2B). We then examined expression of other Toll-like receptors (TLRs) and found that these too correlated positively with CD274 (Figure S2C–J). Because several TLRs signal through MyD88 following formation of the supramolecular organizing center [16,17], we also assessed whether CD274 expression correlated with MYD88 expression and again discovered a positive correlation (Pearson r = 0.42, *p* < 0.0001; Figure S2K). We next evaluated RNAseq data from our panel of HNSCC cell lines to determine if these models recapitulated the trends seen in human tumors. Importantly, although we did not observe a correlation between TLRs and CD274 expression in this small dataset, we did identify a correlation between MYD88 and CD274 expression (*n* = 43; Pearson r = 0.50, *p* < 0.001; Figure S2L).

**Figure 3:**
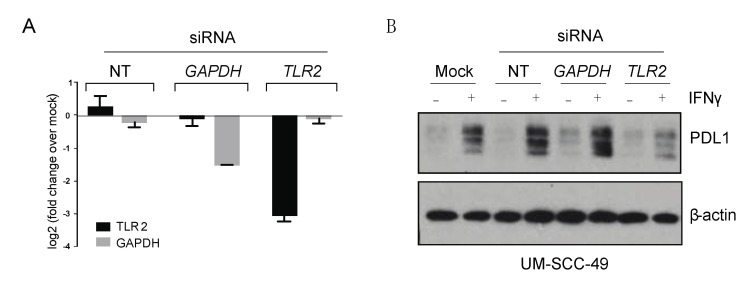
TLR2 depletion attenuates IFNγ-mediated upregulation of PDL1 in UM-SCC-49 cells. (**A**) UM-SCC-49 cells were treated with siRNA as indicated, and knockdown of TLR2 and GAPDH RNA expression was tested by qPCR. (**B**) Cells were transfected with non-targeting (NT), GAPDH, or TLR2 siRNA for 24 h were then treated −/+ 10 ng/mL IFNγ for 48 h, and protein expression was assessed by immunoblot as indicated. The uncropped Western blots have been shown in Figure S3.

While these observations suggest a relationship between TLR2 and PDL1 expression, it is also possible that TLR2 upregulation and PDL1 expression are simply co-occurring features reflecting elevated immune activity in the tumor microenvironment, as PDL1 expression is commonly increased in inflamed tissues [18]. Thus, we sought to establish a causal link between TLR2 and PDL1 in cell line models to directly validate results of the CRISPR screen. To test if modulation of TLR2 expression can influence PDL1 protein expression, TLR2 transcript was knocked down via siRNA (Figure 3A), and changes to total PDL1 protein expression in the presence or absence of IFNγ were assessed. Consistent with our CRISPR screen, we observed a decrease in the ability of IFNγ to induce PDL1 total protein expression when TLR2 RNA was depleted, demonstrating that our approach successfully identified a gene capable of regulating PDL1 expression in UM-SCC-49 (Figure 3B).

**Figure 4:**
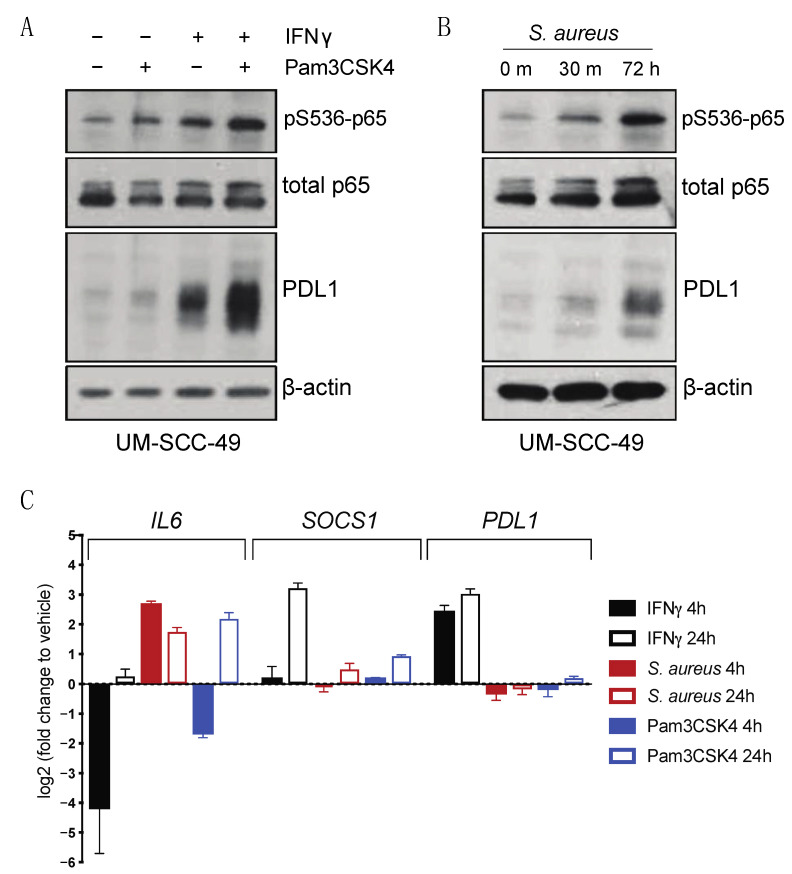
TLR2 agonists drive PDL1 protein accumulation in UM-SCC-49 cells. (**A**) UMSCC49 cells were treated with or without 300 ng/mL Pam3CSK4 (Invivogen) with or without 10 ng/mL IFNγ for 72 h. (**B**) UM-SCC-49 cells were treated for indicated duration with 0.075% S. aureus (Millipore) and lysates were analyzed by immunoblot. (**C**) qPCR analysis of IFNγ and TLR effectors and PDL1 expression in UM-SCC-49 treated with S. aureus or Pam3CSK4 for either 4 h or 24 h as indicated.

We then sought to test whether activation of TLR2 in our cell line models could induce significant changes in either baseline or IFNγ-induced PDL1 expression. Pam3CSK4 is a synthetic lipopeptide that acts as a specific TLR1/2 agonist [19]. Wild-type UM-SCC-49 and UM-SCC-92 cells were treated with Pam3CSK4 for 72 h and a moderate increase in total PDL1 protein expression was observed. However, when Pam3CSK4 was given concurrently with IFNγ, the ability of IFNγ to induce PDL1 expression was enhanced (Figure 4A and Figure S4). Because activation of TLR2 is known to induce phosphorylation and activation of the p65 subunit of NFκB (also referred to as RelA) [20], we also assessed serine 536 phosphorylation of p65. We observed increased phosphorylated p65 in response to Pam3CSK4 or IFNγ alone, with a further increase when both factors were present (Figure 4A).

Next, we considered the role of TLR2 in initiating an immune response. TLRs recognize pathogen associated molecular patterns (PAMPs) and initiate signaling cascades to drive various aspects of immune response, including production of pro-inflammatory cytokines [15]. Given that HNSCC often arises in tissues with high exposure to pathogens, we postulated that TLR2 may modulate PDL1 expression following activation by PAMPs through enhanced NFκB signaling. Previous studies have shown that bacteria such as *Staphylococcus aureus* (*S. aureus*) both activates TLR2 and induces PDL1 expression in monocytes [21]. *S. aureus* is also prevalent in the oral microbiomes of oral cancer patients [22]. UM-SCC-49 cells were therefore treated with heat-inactivated *S. aureus,* and induction of both p65 phosphorylation and total protein expression of PDL1 was observed (Figure 4B). We next analyzed IFNγ-regulated (SOCS1) [23] and p65-regulated (IL6) [24] effectors and showed that both Pam3CSK4 and *S. aureus* induced p65 target IL6 as expected, but interestingly, there was minimal induction of PDL1 transcript expression in the absence of IFNγ. This suggested that IFNγ appears to induce a distinct transcriptional profile at these timepoints, including the unique regulation of known IFNγ target genes such as SOCS1 (Figure 4C and Figure S5).

**Figure 5:**
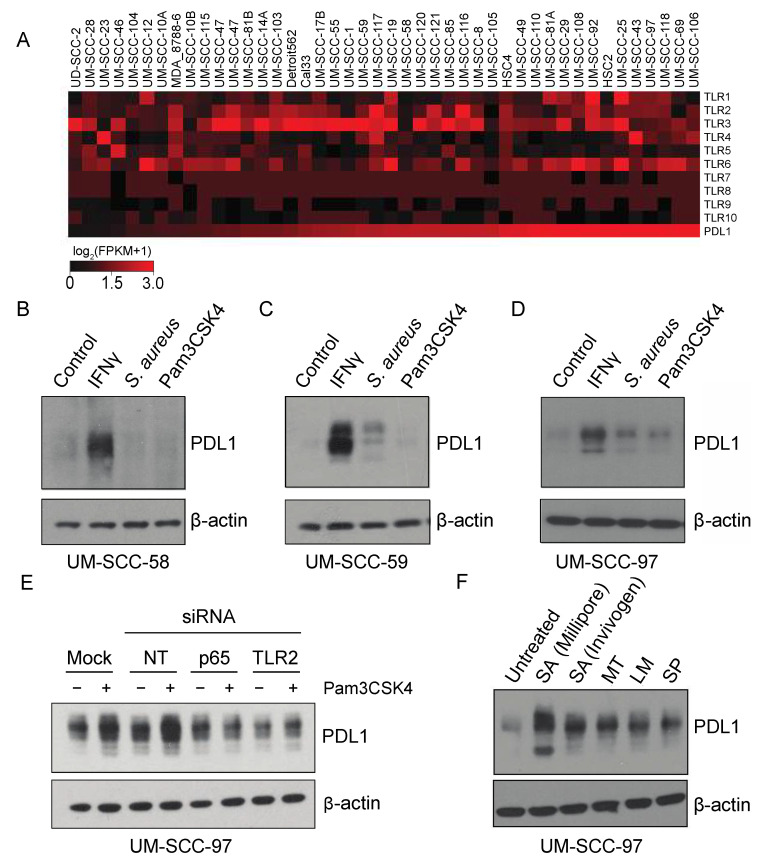
PAM4CSK4-mediated PDL1 stimulation is TLR2-dependent in HNSCC cell lines. (**A**) RNA expression (log_2_(FPKM+1)) of indicated genes in HNSCC cell lines. RNA sequencing was performed for 43 HNSCC cell lines using Illumina stranded transcriptome library kits, as described in (Mann et al.) Heatmaps were generated using MeV software version 4.9 based on log_2_(FPKM+1) values. Cell lines are arranged from left to right in order of increasing expression of PDL1 (CD274). (**B**–**D**) HNSCC cells were treated with vehicle control, 10 ng/mL IFNγ, 0.075% S. aureus (Millipore), or 300 ng/mL Pam3CSK4 for 72 h. PDL1 expression was analyzed by immunoblot. (**E**) UM-SCC-97 cells were transfected with indicated siRNAs. 24 h post transfection, cells were treated with Pam3CSK4 for an additional 48 h. PDL1 was analyzed by immunoblot. (**F**) UM-SCC-97 was incubated with heat-killed commercially available bacterial strains as indicated for 72 h. Abbreviations: NT, Non-Targeting siRNA; SA, *S. aureus*; MT, *Mycobacterium tuberculosis;* LM, *Listeria monoytogenes;* SP, *Streptococcus pneumoniae*.

To examine the ability of Pam3CSK4 and *S. aureus* to regulate PDL1 in other HNSCC models, we chose to advance models from our panel of 43 cell lines based on relative expression of the Toll-like receptors as determined by RNAseq, as TLR2 is thought to heterodimerize with TLR1 or TLR6, and because these TLRs are thought to be largely responsible for recognition of bacterial cell wall components [25]. Thus, we selected cell lines for further evaluation based on a summed expression of TLR1, TLR2, and TLR6 RNA, and chose to advance UM-SCC-58 (low expression) and UM-SCC-97 and UM-SCC-59 (high expression) for further evaluation (Figure 5A). UM-SCC-58 showed no increase in PDL1 expression in response to *S. aureus* or Pam3CSK4 (Figure 5B–D). UM-SCC-59 appeared to upregulate PDL1 in response to *S. aureus*, but not the TLR2 specific agonist Pam3CSK4, suggesting that a different receptor may mediate this response. In contrast, UM-SCC-97 upregulated PDL1 in response to both *S. aureus* and Pam3CSK4. Collectively, this suggested that different cell line models exhibit a range of sensitivity to *S. aureus* and Pam3CSK4 stimulation of PDL1 expression.

Finally, given the ability of UM-SCC-97 to strongly upregulate PDL1 in response to TLR2 agonists, we tested whether Pam3CSK4-mediated PDL1 induction was dependent upon TLR2 or p65 expression. UM-SCC-97 cells were transfected with siRNA targeting TLR2, RELA (p65), or controls, and stimulated with either vehicle or Pam3CSK4. Pam3CSK4 did not induce PDL1 expression in either the TLR2 and RELA knockdowns, indicating expression of these genes is required for Pam3CSK4-mediated PDL1 induction (Figure 5E). To further interrogate the role of microbes in PDL1 induction, we tested whether other common commercially available infectious bacteria were sufficient to phenocopy *S. aureus* in the model. Importantly, we found that the *M. tuberculosis, L. monocytogenes* and *S. pneumoniae* bacterial strains were all able to induce PDL1 expression, and although dependency upon TLR2 has not been established in this system, all reportedly activate TLR2 in other settings (Figure 5F) [26,27,28,29]. It is therefore possible that a wide variety of bacterial strains can contribute to an immunosuppressive phenotype in cancer cells.

## 4. Discussion

The present study describes a screening protocol for the discovery of genes and pathways regulating cell surface PDL1. Multiple gRNAs targeting TLR2 pathway genes were enriched in cell populations selected for altered PDL1 expression, suggesting a role for cancer cell-intrinsic TLR2 in PDL1 regulation. We were surprised to note that sequencing of the PDL1^low^ population did not nominate CD274, the gene encoding PDL1, despite the presence of CD274 targeted gRNAs in the unsorted control library. Further investigation would be necessary to understand the cause of this observation, but it is possible that the CD274 targeting gRNAs did not efficiently knockout CD274 in the model, and these data are consistent with recent shifts in the field toward using high density gRNAs to enhance the accuracy of the approach. Despite this limitation, we chose to continue with our analysis pipeline and focus on genes that were highly enriched in the data set.

Our data indicate an ability of HNSCC cells to upregulate PDL1 in response to TLR stimuli, which may be present in the microenvironment. This observation is consistent with previous studies in monocytes showing that *S. aureus* both activates TLR2 and induces PDL1 expression, suggesting pathway conservation between cell types [21]. Further, our data support the postulate that tumors arising in the context of chronic inflammation may be likely to utilize TLR signaling to upregulate and/or sustain PDL1 expression. Numerous studies associate PDL1-positive tumors with chronically inflamed tissue types, such as tonsillar crypts, indicating that these tissues may represent a permissive niche hospitable to tumorigenesis [18]. The present study suggests that engagement of TLRs on tumor cells by bacterial ligands in the microenvironment could further contribute to immune evasion. With numerous clinical trials currently investigating TLR agonists as cancer therapeutics, the impact of TLR activity on immune checkpoint expression on tumor cells may be of particular importance for clinical implementation [30].

Our finding that TLR2 ligands directly promote upregulation of PDL1 in HNSCC models supports future inquiry into the impact of oral microbiome composition on response to immunotherapy and whether this could be influenced by tumor-intrinsic aberrant expression or activity of TLRs. A myriad of roles for specific bacterial taxa in modulating anti-tumor immune responses have been described in melanoma, and the abundance of certain bacteria has been associated with such metrics as survival, response to therapy, and risk of treatment toxicities in human cancers and in murine models [31,32,33]. Generally, it appears that components of the normal gut microbiome confer therapeutic response and survival benefits, while imbalances may lead to detrimental immune inhibitory effects [31,34]. As these studies exclusively consider the gut microbiome, the local and systemic impact of the oral microbiome in HNSCC remains unclear, and the notion that oral bacteria may act directly on tumor cells to modulate immunogenicity has not been thoroughly explored. Recently, periodontal bacteria *Porphyromonas gingivalis* and *Fusobacterium nucleatum* have been implicated in carcinogenesis [22,35]. Both are also known to activate TLR2 [36,37], leading us to speculate that these bacteria might directly contribute to immune evasion in tumor cells expressing TLR2 during oral carcinogenesis.

The possibility that TLR2 may facilitate immunosuppression in tumor cells is also of particular interest due to the clinical advancement of TLR2-based treatments for cancer [38]. The role of TLR2 in cancer appears complex, confounded in part by redundancy of the multiple TLRs and the diversity of cell types expressing them. Studies in other cancers have demonstrated both pro- and anti-tumorigenic roles of TLR2 and other TLRs [20,39,40,41]. TLR2 is upregulated on epithelial cells in many tumor types and may support tumor growth in this context. This hypothesis led to the development of small molecule and antibody antagonists currently in clinical trials [42,43,44]. Interestingly, TLR2 agonists are also thought to suppress tumor growth by acting directly on T cells to stimulate an immune response, and these have also been advanced to clinical trials [45,46]. Further, multiple trials are investigating TLR8 and TLR9 agonists in combination with the biologic cetuximab, which targets EGFR [47,48]. Rationalizing this approach is the observation that cetuximab can induce antibody-dependent cellular cytotoxicity in addition to inhibiting EGFR activity and that this effect could be enhanced by activation of TLRs [48]. The results of our study suggest that the potential for PDL1 upregulation under these conditions could be an important consideration and may support either more scrupulous patient selection for TLR agonist therapy or the addition of PD1 inhibition to this regimen. In any case, understanding the therapeutic implications of TLR2 activity will require disentangling the complex interactions between oral bacteria, cancerous cells, and the local and systemic immune system, and these will likely be context-specific. In vivo modeling will be necessary to fully appreciate the functional significance of our observations.

## 5. Conclusions

Collectively, our data demonstrate a mechanistic link between microbial stimuli and PDL1 in HNSCC. Importantly, we validate a new profiling approach for the discovery of PDL1 regulatory pathways in HNSCC and show that the NFκB pathway plays a role in mediating signal transduction from TLR2 to PDL1. Given the recent clinical successes of immune checkpoint inhibitors in recurrent/metastatic HNSCC, our data prompt many new questions about the role of TLR signaling in response to therapy as well as immune evasion early in carcinogenesis. We anticipate that future studies of the pathway will clarify the potential to leverage the microbiome, TLR signaling, and/or NFκB pathway activity as a biomarker of response to checkpoint inhibition or, possibly, as therapeutic targets to improve overall survival.

## Figures and Tables

**Table 1:** gRNAs ranked by enrichment in PDL1^low^ over control.

Rank	gRNA Target	PDL1^low^/Control	PDL1^high^/Control
1	RNF130	130.4702	0.61437
2	UIMC1	97.62247	0.533461
3	KLHL30	97.47681	0
4	C1orf116	93.29673	0.604938
5	SLC2A6	93.00492	0.467358
6	TLR2	80.91354	0.363622
7	KIDINS220	74.24762	0.376101
8	COMP	73.34039	0.374752
9	STAB1	48.71035	0.181629
10	NFYB	45.65152	0.163268
11	DISP2	38.79179	0.872197
12	SLC25A25	36.74726	0.14868
13	C7orf55	36.6708	0.194452
14	SSMEM1	34.35596	0.153269
15	TSPAN31	30.65758	0.121086
16	UNKL	27.33073	0.127668
17	ITSN1	14.07346	0.076536
18	HIRA	13.86975	0.066925
19	ZDHHC24	11.48397	5.910346
20	NODAL	11.19729	0.06114
21	TTL	7.297645	0.027327
22	C1orf35	7.269804	0.038215
23	ULBP2	6.721698	0.028772
24	AXDND1	6.5365	0.040082
25	ZFP64	6.110201	0.042713
26	MAGEA6	5.625484	0.045438
27	PCDHGB6	5.256831	0.039738
28	FAM168B	4.806231	0.031167
29	FABP6	4.648937	0.03478
30	USP16	4.350071	0.028961
31	VPS25	4.180224	0.033329
32	SPATA31A2	4.175848	0.035267
33	NPR1	3.975242	0.033517
34	CKMT1A	3.801816	0.020674
35	CCDC117	3.520316	0.02719
36	KIF13B	3.131082	0.022894
37	FZR1	2.847764	0.022893
38	ZNRF3	2.696549	0.02445
39	STRN3	2.642702	0.026301
40	BLCAP	2.598926	0.020274
41	GMNC	2.586674	0.020701
42	KDM6A	2.56034	0.026508
43	OR5T2	2.554679	0.028434
44	CLNS1A	2.489466	0.021168
45	RAD50	2.088789	0.0218049
46	NARR	2.070283	0.020284
47	HINT1	2.068037	0.018824
48	IRG1	2.043546	0.0089151
49	SLC9A6	2.043088	0
50	TTC34	1.554888	0.022822

**Table 2:** gRNAs ranked by enrichment in PDL1^low^ over control.

Rank	gRNA Target	PDL1^high^/Control	PDL1^low^/Control
1	IZUMO3	223.6348	1.0669
2	hsa-mir-105-1	167.3409	1.178067
3	GLS	111.713	0.854239
4	FAT2	104.4253	0.590414
5	SCGB3A1	50.81308	0.337907
6	RAPGEF3	37.84282	0.242991
7	AP2A1	31.35016	0.214096
8	TRIM49	29.33133	0.181142
9	hsa-mir-105-2	9.937754	0.060283
10	FIGLA	6.562241	0.021861
11	POGLUT1	6.558918	0.06121
12	ZDHHC24	5.910346	11.48397
13	KCNIP2	5.604062	0.0485690
14	ZNF37A	4.446165	0.038013
15	TTC5	3.348804	0
16	SLC35F3	2.39854	0
17	FAM71C	2.325857	0
18	CXorf21	2.179963	0.014468
19	RBBP8	2.111634	0.04188
20	CCDC129	1.992549	0
21	SLC5A8	1.902974	0
22	CHST2	1.864696	0.068597
23	P2RY2	1.836203	0
24	SEMA6C	1.834026	0.015726
25	SPHK1	1.797793	0
26	KLHL30	1.794423	0.007607
27	hsa-mir-212	1.77299	0
28	CARNS1	1.744393	0
29	BRF2	1.744393	0
30	LMNB2	1.744393	0
31	MCL1	1.744393	0
32	GNA13	1.744393	0
33	NF2	1.722029	0.714116

**Table 3:** Log_2_(RSEM+1) values from TCGA Head and Neck Cancer cohort (*n* = 566) were retrieved from the UCSC cancer genomics browser (xenabrowser.net).

Gene Set	Gene	Pearson’s R	Adjusted p-Value
***gRNAs enriched in PDL1^low^***	RNF130	0.082815	NS
	UIMC1	0.136287	0.03342197
	KLHL30	0.046933	NS
	C1orf116	0.033465	NS
	SLC2A6	0.143694	0.01757946
	TLR2	0.324183	0.01798496
	KIDINS220	0.143437	NS
	COMP	−0.004867	1.13 × 10^−11^
	STAB1	0.29879	0.00021754
	NFYB	−0.187014	NS
***gRNAs enriched in PDL1^high^***	GLS	0.137618	0.02984585
	FAT2	0.242119	1.56 × 10^−7^
	SCGB3A1	−0.22681	1.42 × 10^−6^
	RAPGEF3	−0.023542	NS
	AP2A1	−0.159187	0.00414437
	TRIM49	−0.093222	NS
***TLR and IFNγ pathway genes***	STAT1	0.60212877	1.15 × 10^−55^
	JAK2	0.69457561	3.05 × 10^−81^
	TLR2	0.32418282	7.46 × 10^−14^
	TLR1	0.30202603	6.12 × 10^−12^
	TLR3	0.44556113	1.71 × 10^−27^
	TLR4	0.39553731	3.55 × 10^−21^
	TLR5	0.08243978	NS
	TLR6	0.28567334	1.25 × 10^−10^
	TLR7	0.41385687	2.29 × 10^−23^
	TLR8	0.53208367	3.15 × 10^−41^
	TLR9	0.17934421	0.00051316
	TLR10	0.27146896	1.48 × 10^−9^
	MYD88	0.42093739	2.99 × 10^−24^
	RELA	−0.0036575	NS

Correlations between *CD274* (PDL1) and individual genes targeted by the top ten most enriched gRNAs in PDL1^low^ and PDL1^high^ were calculated using Pearson r test. Gene expression data for *IZUMO3,* hsa-mir-105-1, and hsa-mir-105-2 were not available. Data for the gene *FIGLA* were excluded as only 15/566 samples had expression values ≥1.

## Data Availability

Data is contained within the article or supplementary material. Gene expression data from RNA-seq experiments are available in the NCBI GEO through GEO Series accession # GSE126975.

## Supplementary Materials

The following are available online at https://www.mdpi.com/article/10.3390/cancers13194782/s1, Figure S1: Dysregulated PDL1 expression in monoclonal cell lines isolated from PDL1^low^ sorted population; Figure S2: RNA expression in TCGA HNSC cohort, Figure S3: Uncropped western blot images, Figure S4: Pam3CSK4 and S. aureus enhance IFNγ-induced PDL1 expression, Figure S5: Expression of IFNγ and NFκB target genes, Table S1: PCR primers for GeCKO library preparation, Table S2: Primer sequences for qPCR.
