# Supplementary material for: Microbe-Mediated Activation of Toll-like Receptor 2 Drives PDL1 Expression in HNSCC
Source: Cancers (Basel). 2021 Sep 24;13(19):4782. doi: 10.3390/cancers13194782 (PMC8508280; doi:10.3390/cancers13194782)
Supplement: Supplementary file 1 [file cancers-13-04782-s001.zip › cancers-1326428-supplementary.pdf]

cancers

MDPI

Supplementary Materials: Microbe-Mediated Activation of Toll-like Receptor 2 Drives PDL1 Expression in HNSCC

Jacqueline E Mann, Megan L Ludwig, Aditi Kulkarni, Erin B Scheftz, Isabel R Murray, Jingyi Zhai, Elizabeth Gensterblum-Miller, Hui Jiang and J Chad Brenner

Table S1. PCR primers for GeCKO library preparation.

		Primer Sequence
Reaction #1	F	AATGGACTATCATATGCTTACCGTAACTTGAAAGTATTTCG
	R	GGTCTTGAAAGGAGTGGGAATTGGCTCCGGTGCCCGTCAG
Reaction #2	F	AATGATACGGCGACCACCGAGATCTACACTCTTTCCCTACACGACGCTCTTCCGATC T(1-9bp stagger)AAGTAGAGtcttgtggaaaggacgaaacaccg.
	R	CAAGCAGAAGACGGCATACGAGATTCGCCTTAGTGACTGGAGTTCAGACGTGTGCT CTTCCGATCTataacggactagccttattttaac

The underlined sequence in the Forward primer indicates a representative 8 base pair barcode, which was different for each sample (control, PDL1high, PDL1low). Uppercase sequence indicates Illumina adapters. The forward primer contains the TruSeq Universal adapter, and the reverse primer consists of Illumina P7, 8 base pair index, and multiplexing PCR primer 2.0. Lowercase sequence indicates the priming sites for the lentiviral construct. F, forward primer; R, reverse primer.

Table S2. Primer sequences for qPCR.

Target	Forward (5′-3′)	Reverse (5′-3′)
myd88	GACTGCTCGAGCTGCTTACC	ACATTCCTTGCTCTGCAGGT
TGFb1	CGACTCGCCAGAGTGGTTAT	CGGTAGTGAACCCGTTGATGT
tlr1	GCATATTGGGCACCCCTACA	TAGGAACGTGGATGAGACCG
tlr6	CCGTTCATTTACAAGTTATTTTCTC	TGTTGCAGTGGCTATCCTAA
nfkb1	CGCTTAGGAGGGAGAGCCC	GCAGTGCCATCTGTGGTTGA
tnf	ACTTTGGAGTGATCGGCCC	ATTGGCCAGGAGGGCATTG
irf7	GCCTGGCCACCATAAAAGCG	TGTTGAACCAGTGTCCAGGC
rela	CGCATCCAGACCAACAACAA	TTGGGGGCACGATTGTCAAA
gapdh	AATGGGCAGCCGTTAGGAAA	GCCCAATACGACCAAATCAGAG

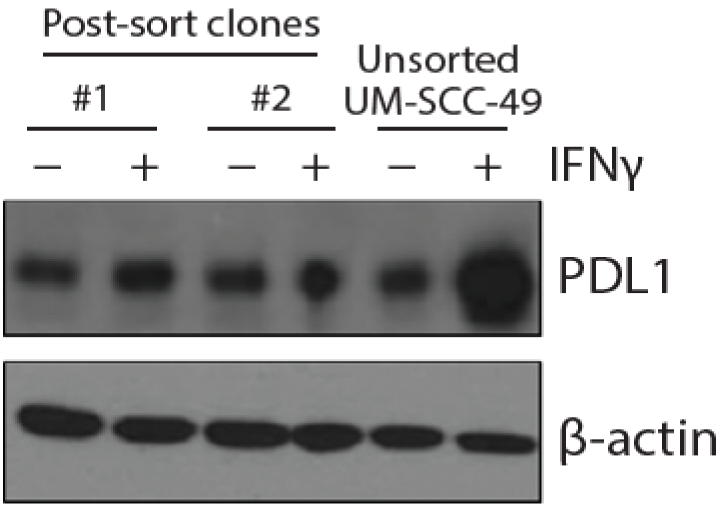

Figure S1. Dysregulated PDL1 expression in monoclonal cell lines isolated from PDL1^{low} sorted population. Cell lines were arbitrarily designated Clone #1 and #2. Cells were treated +/- 10 ng/mL interferon-gamma for 72 h and PDL1 expression was assessed by immunoblot.

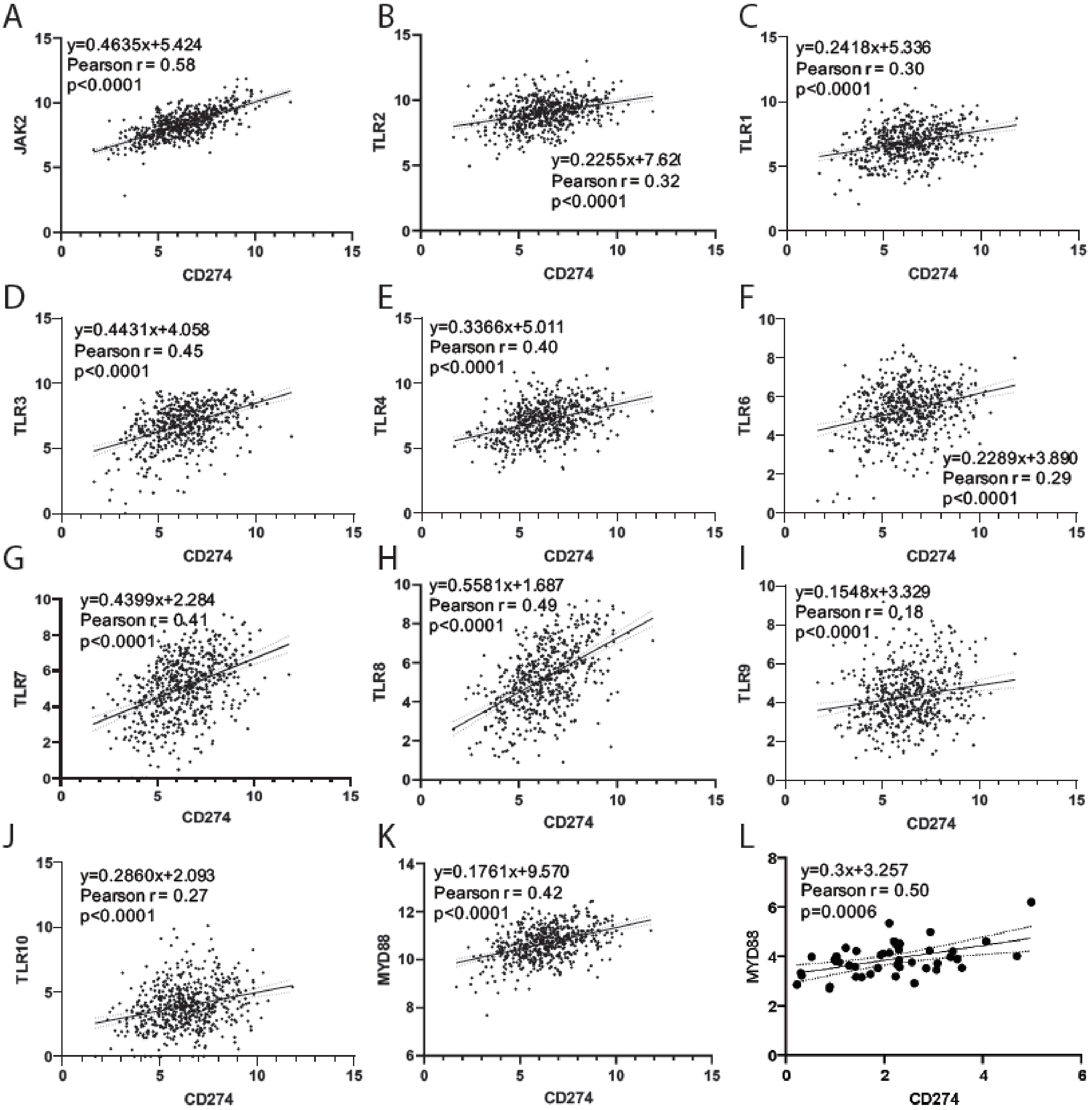

Figure S2. RNA expression in TCGA HNSC cohort. A-K) Log2(RSEM+1) values from TCGA Head and Neck Cancer cohort (*n* = 566) were retrieved from the UCSC cancer genomics browser (xenabrowser.net). Correlations. between genes of interest (y-axis) and PDL1 (*CD274;* x-axis) were calculated using Pearson r test and plots were generated in GraphPad Prism 8 software. L) Log2(FPKM+1) values for PDL1 (*CD274*) and MYD88 RNA from HNSCC cell lines (*n* = 43) were analyzed as above.

Figure 3B

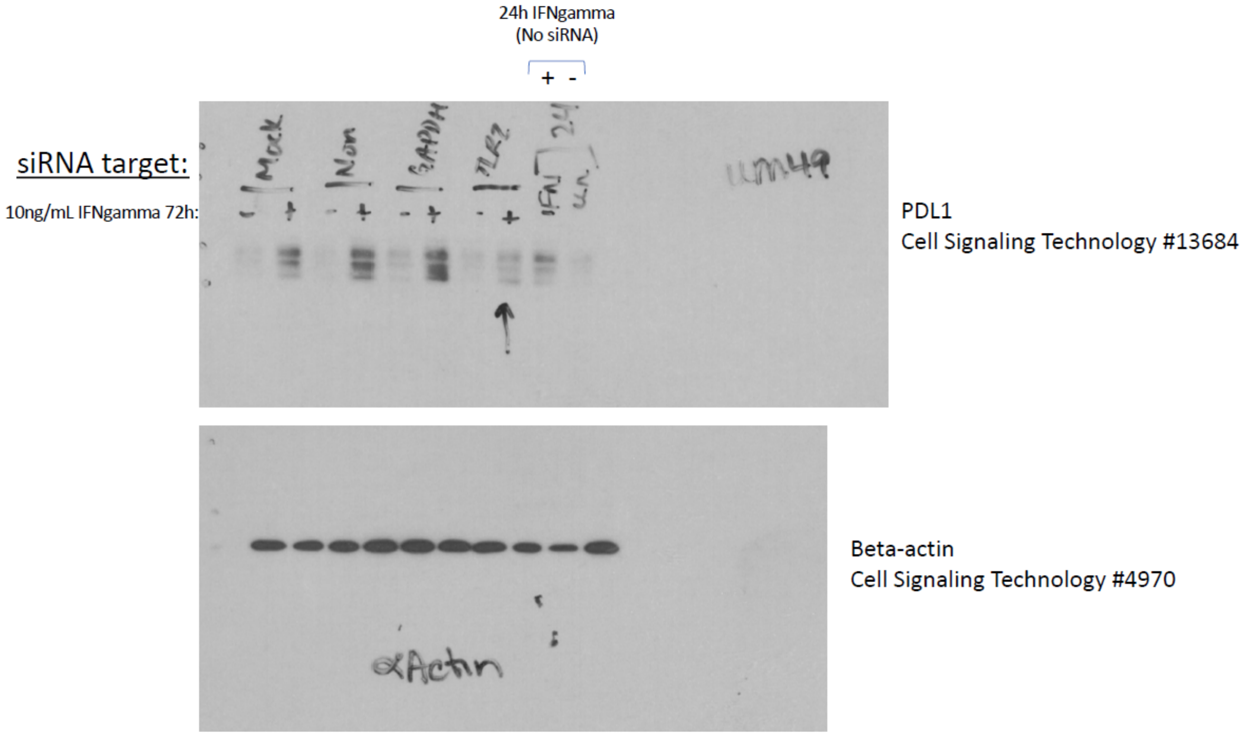

Figure 4A

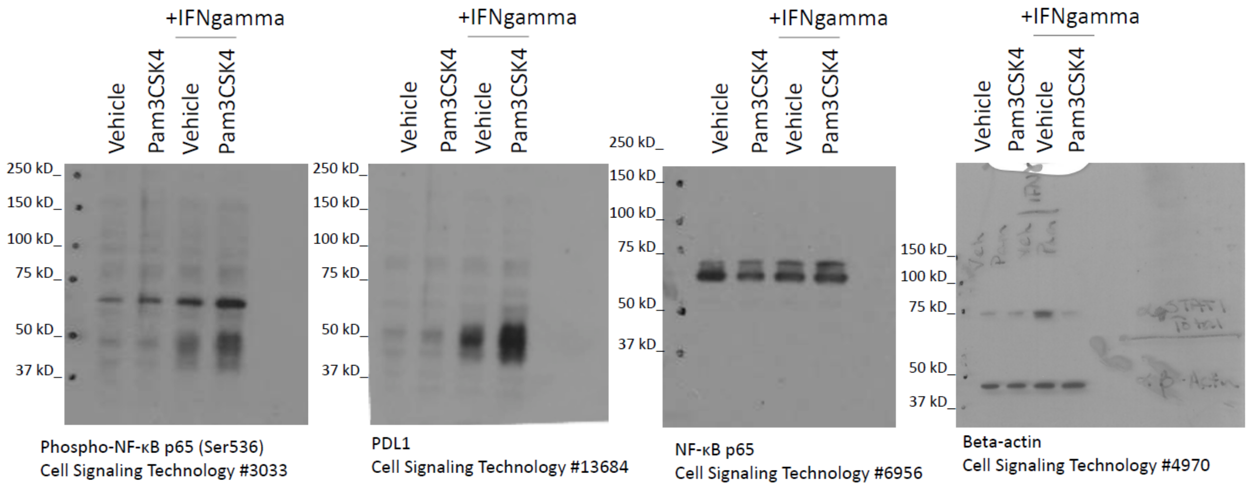

Figure 4B

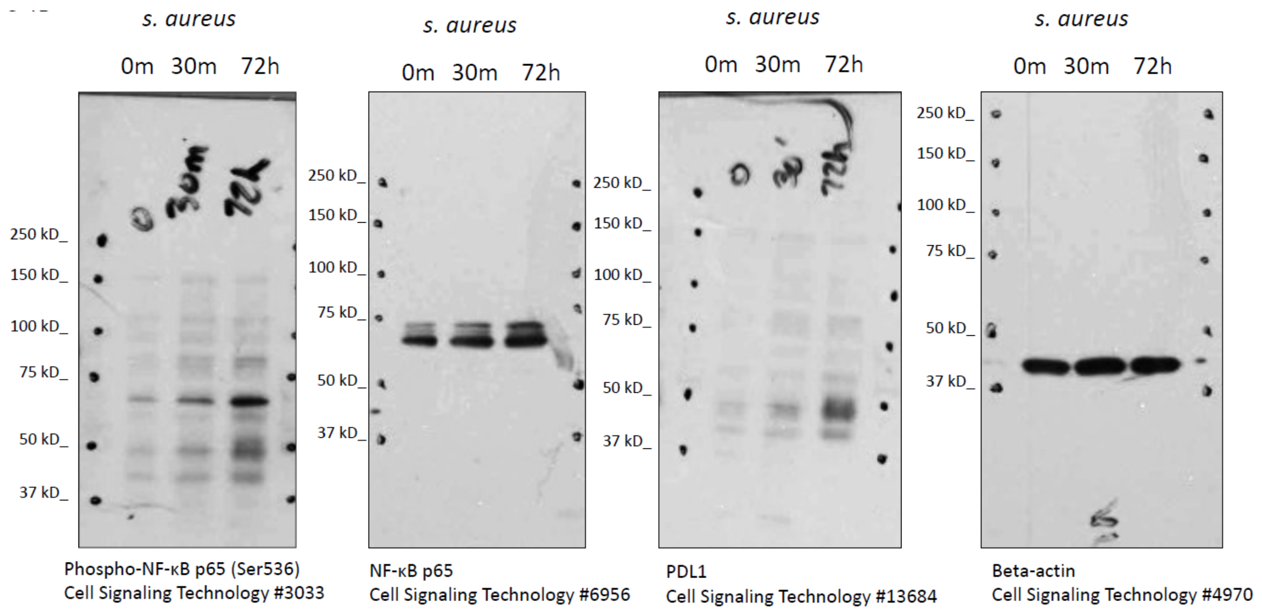

Figure 5B,C

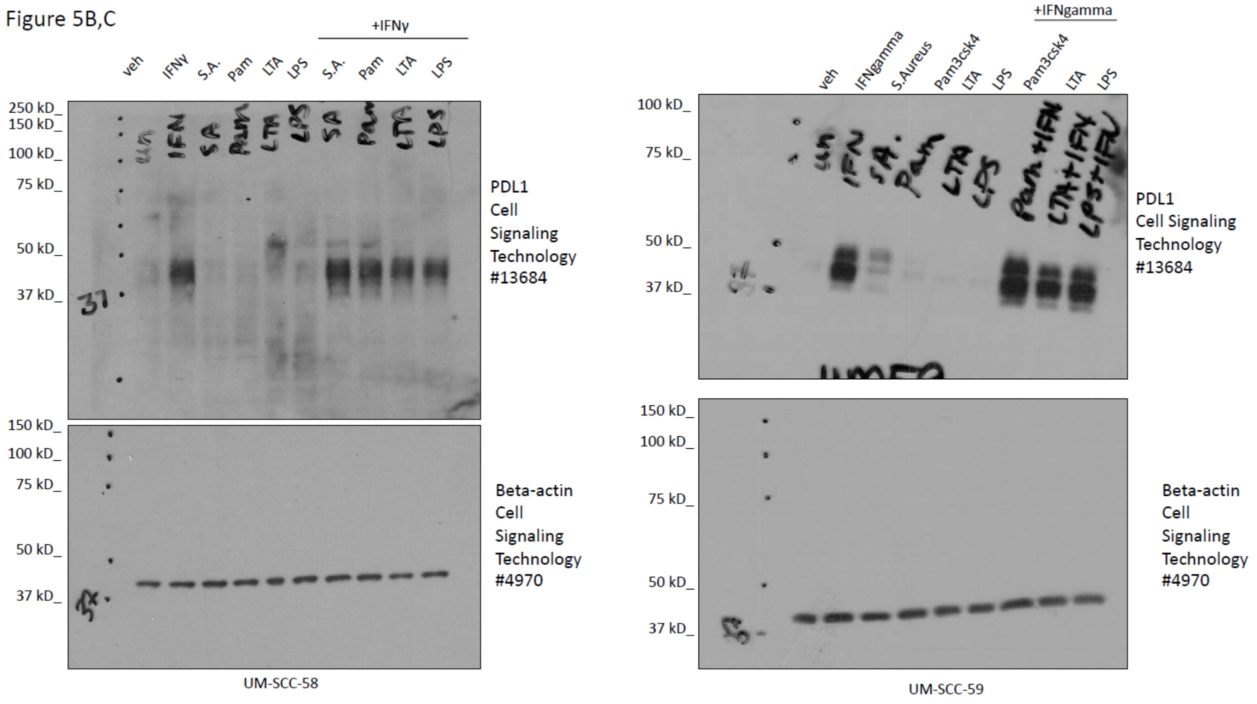

Figure 5D

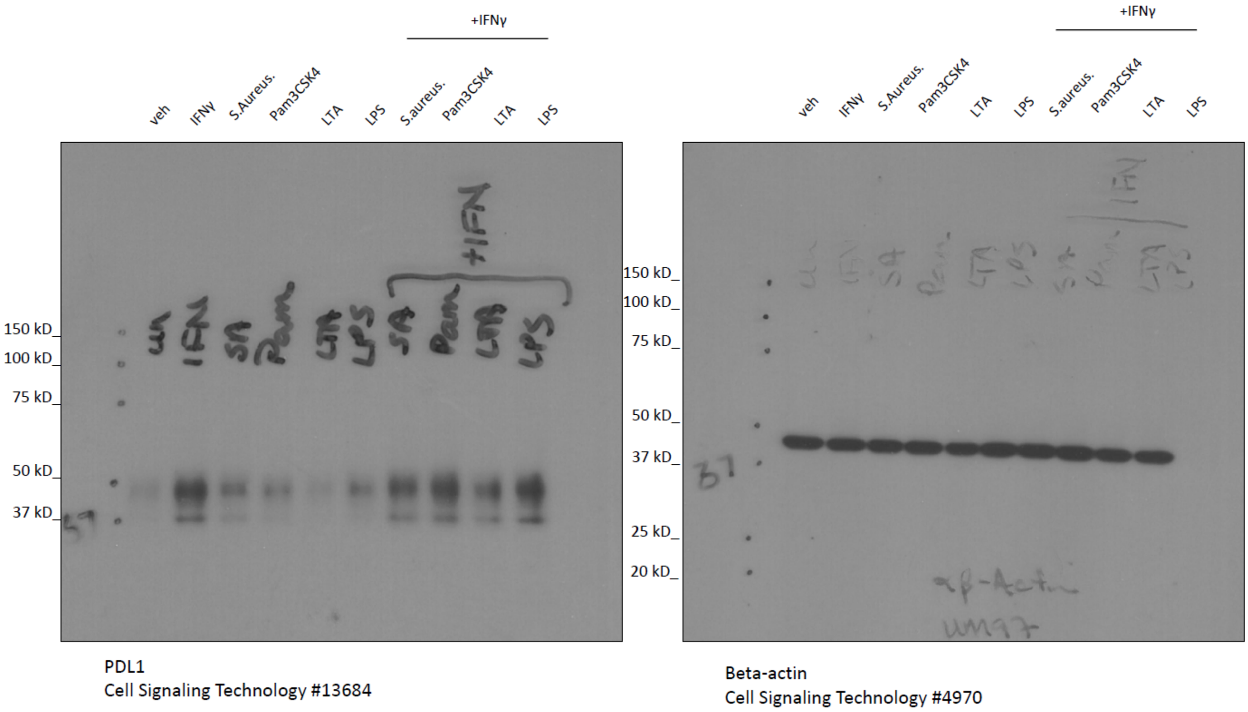
+IFNγ
veh
IFNγ
S.Aureus.
Pam3CSK4
LTA
LPS
S.aureus.
Pam3CSK4
LTA
LPS
150 kD
100 kD
75 kD
50 kD
37 kD
25 kD
20 kD
PDL1
Cell Signaling Technology #13684
Beta-actin
Cell Signaling Technology #4970

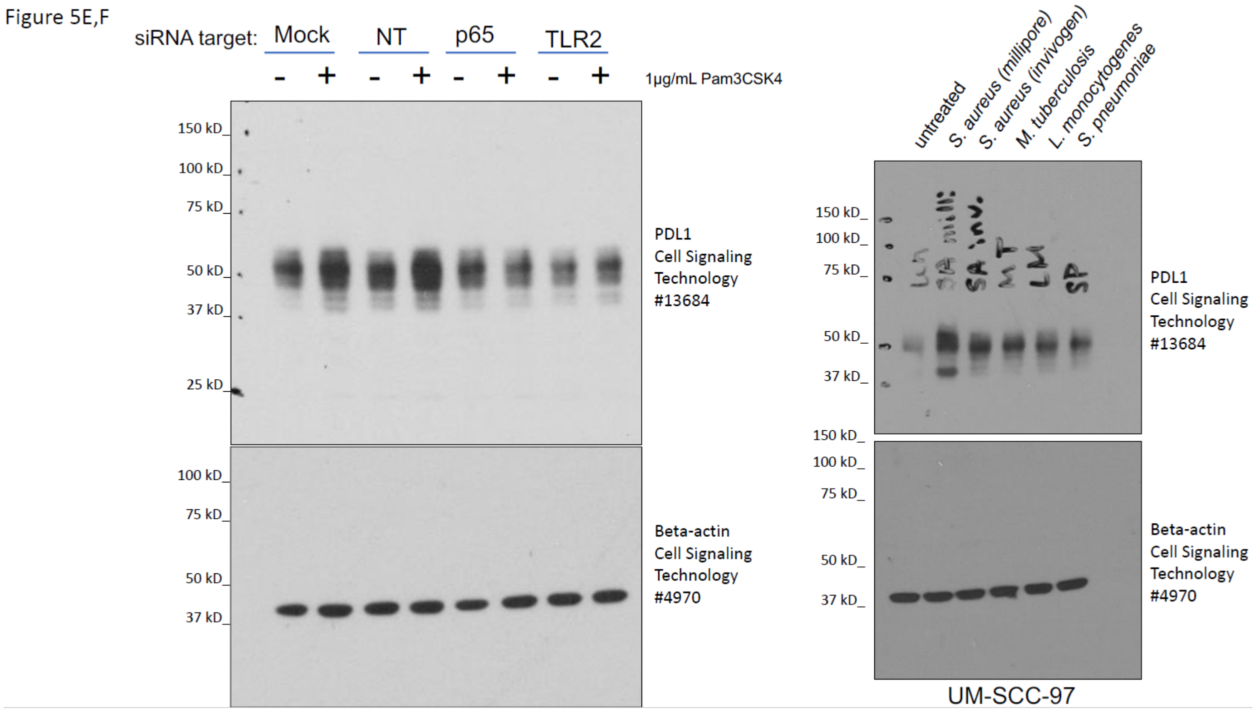
Figure 5E,F
siRNA target: Mock NT p65 TLR2
- + - + - + - +
1µg/mL Pam3CSK4
150 kD
100 kD
75 kD
50 kD
37 kD
25 kD
PDL1
Cell Signaling Technology #13684
Beta-actin
Cell Signaling Technology #4970
untreated
S. aureus (millipore)
S. aureus (invivogen)
M. tuberculosis
L. monocytogenes
S. pneumoniae
UM-SCC-97

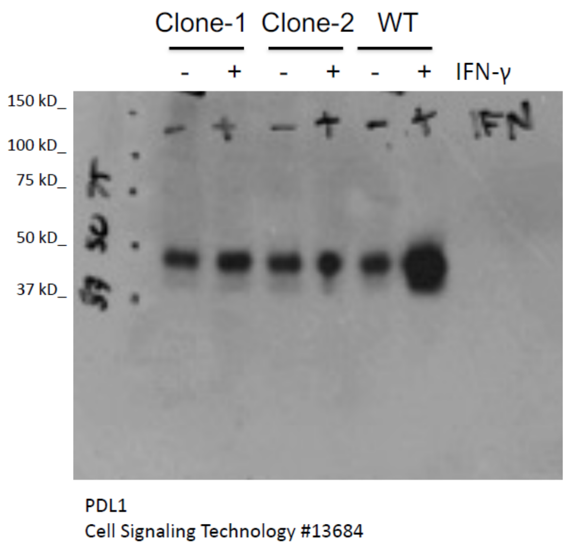

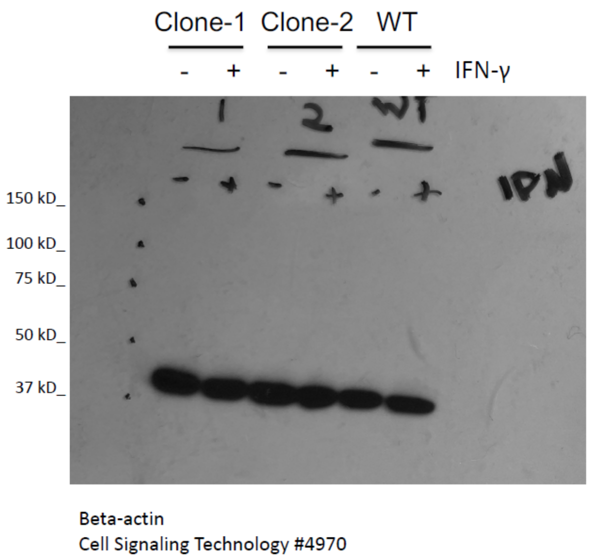

Figure S3. Uncropped western blot images.

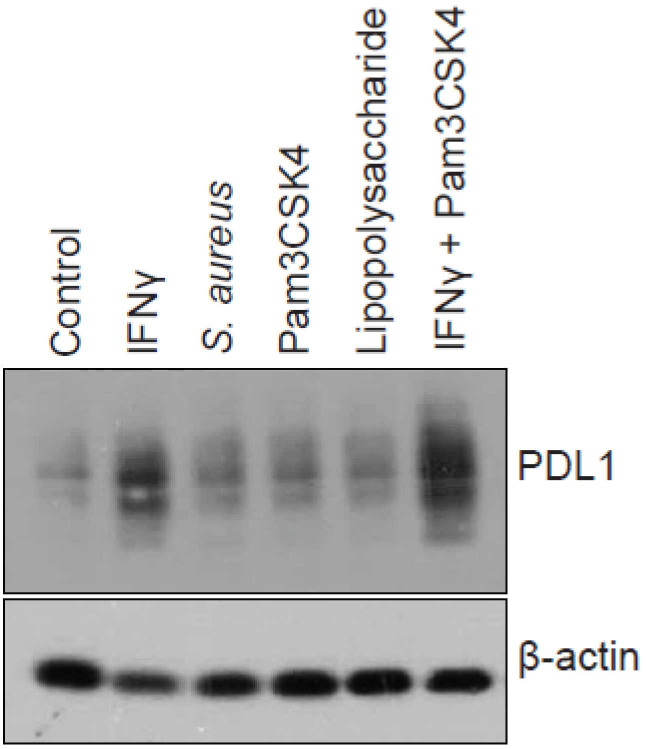

Figure S4. Pam3CSK4 and *S. aureus* enhance IFNγ-induced PDL1 expression.

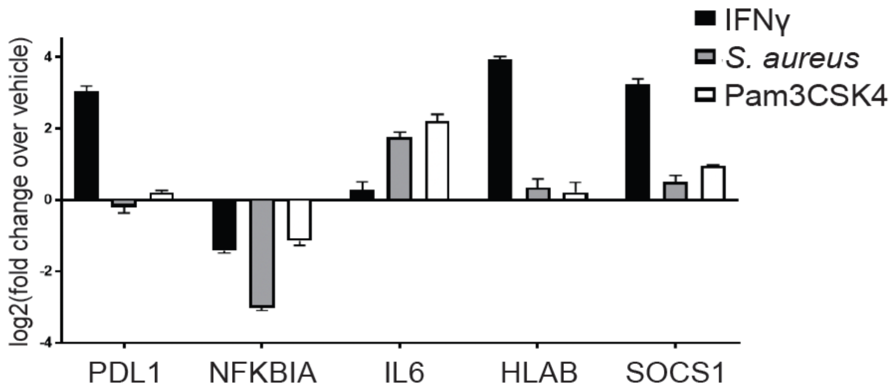

Figure S5. Expression of IFNγ and NFκB target genes, UM-SCC-49 cells were treated as indicated for 72 h. qRT-PCR was performed.
